# Do Tree Size and Tree Shade Tolerance Affect the Photosynthetic Capacity of Broad-Leaved Tree Species?

**DOI:** 10.3390/plants12030523

**Published:** 2023-01-23

**Authors:** Yuhan Song, Guangze Jin

**Affiliations:** 1 Center for Ecological Research, Northeast Forestry University, Harbin 150040, China; 2 Key Laboratory of Sustainable Forest Ecosystem Management-Ministry of Education, Northeast Forestry University, Harbin 150040, China; 3 Northeast Asia Biodiversity Research Center, Northeast Forestry University, Harbin 150040, China

**Keywords:** mixed broad-leaved Korean pine forest, photosynthesis, shade tolerance, tree size, woody plants, functional traits

## Abstract

(1) Background: leaf structure traits are closely related to leaf photosynthesis, reflecting the ability of trees to obtain external resources in the process of growth. (2) Methods: We studied the morphological, chemical, anatomical, stomatal traits and maximum net photosynthetic rate of six broad-leaf species in northern temperate mixed broad-leaved Korean pine (*Pinus koraiensis*) forest. (3) Aim: To investigate whether there are differences in leaf structural traits of trees with different shade tolerances and different sizes and the effects of these differences on leaf photosynthetic capacity. (4) Results: the effects of leaf structure traits on leaf photosynthesis were different among trees with different shade tolerances or different sizes. Under the condition of light saturation, the net photosynthetic rate, nitrogen use efficiency, phosphorus use efficiency and stomatal conductance of shade-intolerant trees or small trees were higher than those of shade-tolerant trees or large trees. (5) Conclusions: the shade tolerance of tree species or the size of trees affect the traits of leaf structure and indirectly affect the photosynthetic ability of plants. When constructing the leaf trait–photosynthesis model, the shade tolerance and tree size of tree species should be taken into account.

## 1. Introduction

The essence of the trait differences of different trees in different life history stages is the trade-off in the capacity of trees to obtain light, nutrients, water and other resources under specific environments and physiological conditions to better grow and reproduce [1,2]. Photosynthesis is one of the most important functions of trees. The construction of an appropriate photosynthesis model is helpful for us to predict the photosynthetic capacity of trees under different conditions. As the most important organ of tree photosynthesis, the structural traits of leaves are closely related to tree photosynthetic capacity [3]. In the past few decades, some studies have explored the relationship between leaf photosynthetic capacity and different leaf structure traits [4,5,6]. At the same time, some studies have shown that tree shade tolerance or tree size affect the distribution of leaf resources among structural traits [7,8,9]. However, few studies have linked tree shade tolerance or tree size, leaf structure traits and photosynthesis to explain the supporting mechanism of shade tolerance on plant photosynthesis. The relationship between the structure and function of plant leaves is a research hotspot for ecologists in recent years. The establishment of the relationship model between leaf structure and function is helpful for us to understand the forest ecosystem. Previous studies have pointed out the relationship between some structural traits and the photosynthetic capacity of leaves. For example, some studies have shown that the photosynthetic capacity of leaves is related to the content of nitrogen (N) in leaves. Higher N in leaves usually means that trees can allocate more N to photosynthesis-related enzymes, so plants with higher N in leaves tend to have higher photosynthetic rates [10,11,12]. Some studies have also constructed a model to estimate the carboxylation rate of the Rubisco enzyme using leaf N, so as to predict the photosynthesis rate of trees [13,14]. However, plant functional traits are affected not only by single structural traits, but also by the synergistic regulation of multiple structural traits [15]. N in leaves is not the only factor affecting leaf photosynthetic capacity. Many studies have shown that many structural characters of plant leaves are closely related to the photosynthetic ability of plant leaves [10,16,17]. The most representative theory is the leaf economic spectrum (LES) theory put forward by Wright et al. in 2004, which holds that trees with higher photosynthetic rates are located at the end of resource acquisition and tend to have higher leaf N, leaf phosphorus content (P) and lower leaf mass per unit area (LMA) [17]. Over the next twenty years, this trade-off reflected by LES theory has been verified by a large number of reports and widely recognized [18,19]. However, some structural traits not included in LES are also closely related to photosynthesis. For example, leaf photosynthesis is usually closely related to leaf anatomical structures; thicker palisade tissue promotes a more uniform distribution of light in leaves and usually means more chlorophyll in leaves [20,21], so palisade tissue thickness (PT) tends to be positively correlated with leaf photosynthetic capacity. Plants can better absorb light through the scattering of cells in sponge tissue, so the sponge tissue thickness (ST) of leaves is also closely related to photosynthesis ability [22]. In addition, as the main channel of CO_2_ diffusion, stomata also have an important effect on the photosynthetic ability of leaves. Previous studies have shown that the increase in stomatal conductance (G_s_) can increase the photosynthetic rate of leaves when other restrictions are not significant [23,24]. Understanding and quantifying the relationship between plant leaf traits and the photosynthesis rate is of great significance for the establishment of a plant photosynthesis model. However, for plants, the trait–trait relationship is not constant either between species or within species [1,25]. Therefore, it is necessary to explore the intraspecific and interspecific factors that cause the variation of leaf characters, and to understand the effects of leaf characters on photosynthesis under different conditions.

Within the same tree species, there is often variation in leaf traits. Tree size is one of the decisive factors affecting intraspecific variation [18,26]. Tree size affects the difficulty of obtaining light, water, nutrients and other resources [27] and then changes the relationship between the cost and benefit of plant traits [28]. Therefore, the variation in traits within trees of different sizes reflects the trade-off in resource allocation made by trees for better growth. Previous studies have shown that the change in N and P contents in leaves is a typical characteristic with increasing tree size [29]. Higher growth rates usually require higher N and P contents to maintain [30]. A large amount of resources are allocated to leaves for photosynthesis to promote the rapid growth of young trees; as trees grow, more resources are allocated to stems and roots rather than photosynthetic tissues to enhance competitiveness [1,31,32], resulting in lower N and P contents in leaves of larger trees. Additionally, a previous study showed that after N and P addition, differently sized trees had different responses, in which small trees showed higher growth rates, while the growth rate of large trees did not change [33]. This heterogeneous response to nutrient addition reflects the different nutrient utilization strategies of trees of different sizes. In addition to the differences in chemical traits, there are also differences in the leaf morphological characters of trees of different sizes. Even in the same forest, smaller trees may develop larger or thinner leaves than large trees of the same species, or develop leaves earlier than large trees [34,35] to resist the shade of canopy trees and obtain more light. In addition, there are great differences in stomatal conductance between large trees and small trees. With the growth of trees, the height of the tree leads to an increase in water transport resistance from roots to leaves, which leads to stronger water restriction in large trees, so large trees usually have lower stomatal conductance than small ones, thus reducing their own transpiration rate to cope with water restriction [36,37]. The variation of these leaf structure traits can affect the photosynthetic capacity of trees. Therefore, to explore the effect of leaf traits on plant photosynthesis, we cannot ignore the intraspecific variation in leaf traits with increasing plant size.

The variation of leaf traits among different tree species is mainly caused by genetic differences. Shade tolerance is one of the factors affecting the interspecific variation of leaf traits. The difference of shade tolerance among species reflects the response of species to different light environments [38], determines whether species can grow and reproduce in new habitats [39], and has an important influence on the formation of stand structure in the process of natural forest succession [40,41]. There is evidence that shade-tolerant species tend to have wider crowns and more fixed branching patterns than shade-intolerant species, reducing their self-occlusion and helping them better capture light in low light conditions [42]. The main reason why shade-tolerant species have this crown structure is that their growth environment is often more restricted by light than shade-intolerant species, which makes shade-tolerant species different from shade-intolerant species in leaf traits. For example, in order to make up for the higher construction cost, shade-tolerant trees tend to have longer leaf life to maintain long-term photosynthesis. The leaves of shade-tolerant trees also showed higher mechanical strength, lower N and photosynthetic ability [8,43]. Some studies have described the effects of plant shade tolerance on partial leaf traits [9,44]. However, there are few studies on whether the difference of shade tolerance will lead to the variation of leaf anatomical traits. The formation of PT in leaves usually depends on the light environment of the previous year or the light environment of current mature leaves. Usually, the better the previous light conditions, the higher the PT of new leaves [22]. Therefore, we predict that the PT of shade-tolerant species is lower than that of shade-intolerant species. Previous research also supports the hypothesis that trees with higher PT tend to have better light conditions and can better absorb light [45].

At present, there are few studies on the relationship between tree shade tolerance or tree size, leaf structure traits and photosynthesis ability. In order to explore the mechanism of the effect of tree shade tolerance of tree species or tree size on leaf photosynthesis, we selected six different shade-tolerant broad-leaved tree species in mixed broad-leaved Korean pine (*Pinus koraiensis*) forest in Northeast China, measured their leaf structural traits and maximum net photosynthetic rate in two life cycle stages, and explored the variation of leaf traits and their effects on leaf photosynthetic capacity. We tested two interrelated hypotheses: (1) there were significant differences in leaf traits between trees of different sizes and shade tolerances; (2) tree size and shade tolerance can affect leaf photosynthetic capacity indirectly by affecting leaf structure traits.

## 2. Results

### 2.1. Variations in Leaf Traits of Different Tree Types

Across all tree species, the range of epidermis thickness (ET) was 16.81–40.62 μm, the range of palisade tissue thickness (PT) was 25.58–103.93 μm, and the range of spongy tissue thickness (ST) was 18.94–84.16 μm (Table S1). All anatomical traits except ST were significantly different between shade-tolerant trees and shade-intolerant trees (Table 1). The PT, palisade–spongy tissue ratio (PT/ST) and palisade tissue–leaf thickness ratio (PT/LT) of shade-intolerant trees were significantly higher than those of shade-tolerant trees, while the ET and spongy tissue-leaf thickness ratio (ST/LT) were significantly lower than those of shade-tolerant trees. There was no significant difference in ST between shade-intolerant and shade-tolerant trees (Table 1). The maximum net photosynthetic rate based on mass (P_n_), carbon content (C), nitrogen content (N), utilization efficiency of photosynthetic nitrogen (PNUE), phosphorus content (P), utilization efficiency of photosynthetic phosphorus (PPUE) and stomatal conductance based on mass (G_s_) of shade-intolerant trees were higher than those of shade-tolerant trees, while the SLA of shade-intolerant trees was lower than that of shade-tolerant trees (Table 1).

Tree species had significant effects on all leaf traits (*p* < 0.05); species with strong shade tolerance tended to have lower P_n_, Gs and higher SLA (Figure S1, Table 2). Tree size had significant effects on all leaf traits except PT/ST, PT/LT and ST/LT (*p* < 0.05). The P_n_, specific leaf area (SLA), G_s_, N, P, PNUE and PPUE of small trees were significantly higher than those of large trees, while the ET, PT, ST and C of small trees were significantly lower than those of large trees (Table 2). The interaction between tree species and tree size had significant effects on P_n_, SLA, G_s_, PT, ST, N, P and PNUE (Table 2).

**Figure 1:**
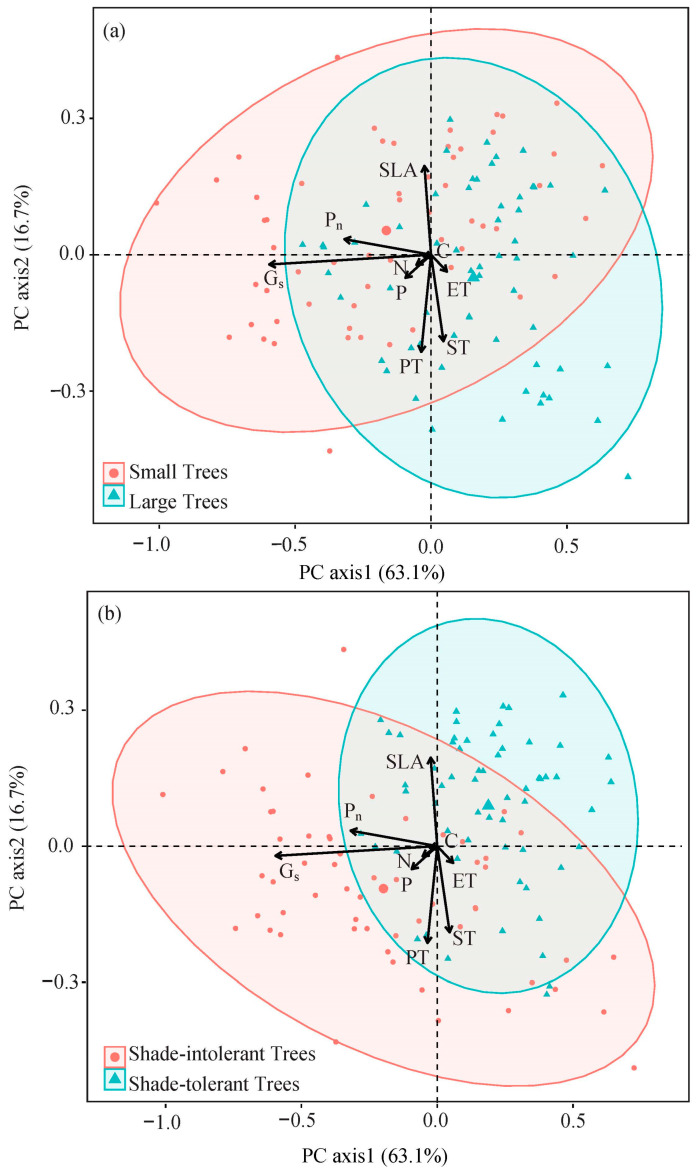
Principal component analysis of 8 leaf traits of different tree sizes or shade-tolerances. The meanings of abbreviations are shown in Table 2. (**a**) Distribution of trees of different sizes with respect to leaf traits; (**b**) Distribution of different shade tolerance trees with leaf traits.

The two principal axes of principal component analysis can explain 63.1% and 16.7% of the total variance, respectively. With the increase in tree size, the leaf traits developed along the direction of SLA decrease, and with the increase in shade tolerance, leaf traits developed along the direction of G_s_ decrease (Figure 1).

### 2.2. Relationship between Leaf Structural Traits and Leaf Net Photosynthetic Rate

**Figure 2:**
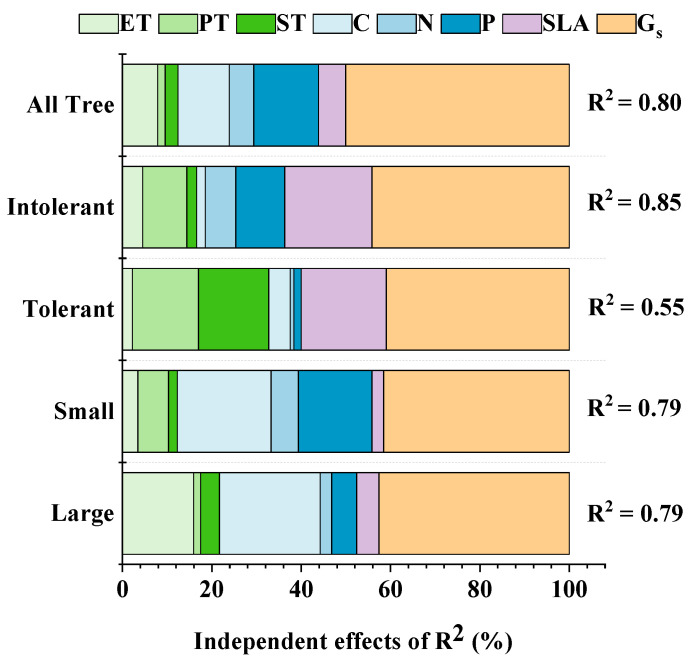
Contribution of leaf traits to P_n_ variation. The meanings of abbreviations are shown in Table 2.

The results of HP analysis showed that although the morphological, anatomical, chemical and stomatal traits of leaves contributed to the variation of P_n_, the contribution rate of G_s_ to the total variation of P_n_ was the largest, which was much higher than that of other traits to P_n_ variation (Figure 2). In different groups, the chemical traits of leaves made a greater contribution to the P_n_ of small trees and shade-intolerant groups, while the anatomical traits of leaves made a greater contribution to the P_n_ of large trees and shade-tolerant species (Figure 2).

**Figure 3:**
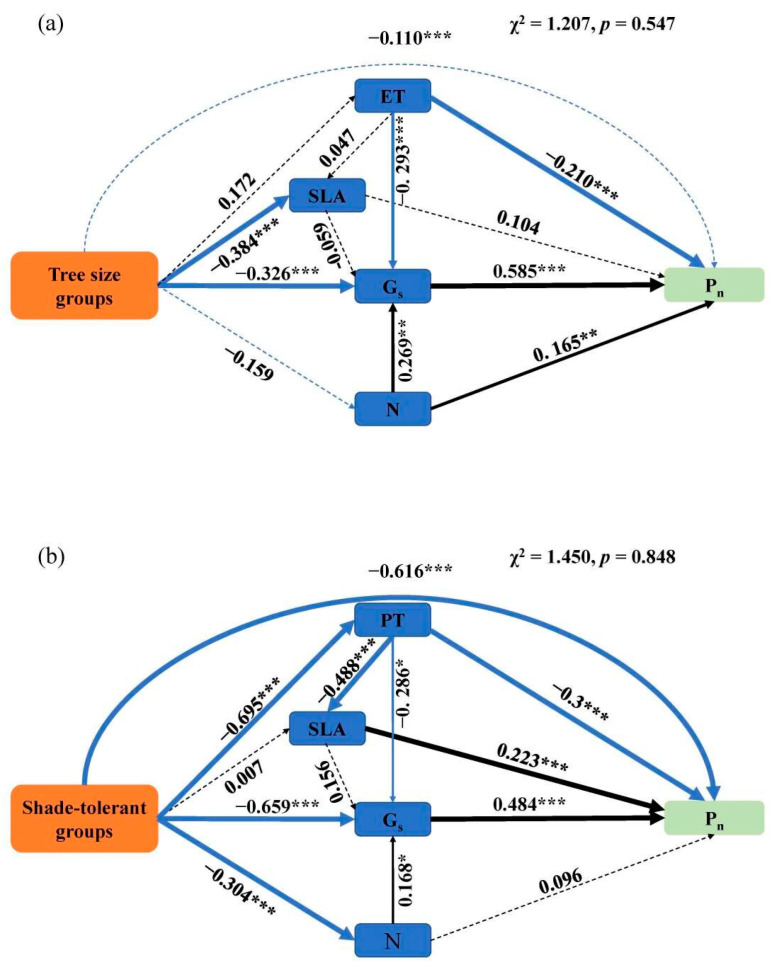
Structural equation model of tree type influencing P_n_. The solid lines indicate significant paths, and the thickness of the solid line represents the degree of significant correlation (*, *p* < 0.05; **, *p* < 0.01; ***, *p* < 0.001.), while the dotted lines indicate nonsignificant paths (*p* > 0.05). The meanings of abbreviations are shown in Table 2. (**a**) Structural equation model of tree sizes influencing P_n_; (**b**) Structural equation model of shade tolerances influencing P_n._

In the SEM, both tree size and shade tolerance had indirect effects on leaf P_n_ through leaf structural traits (Figure 3), while leaf shade tolerance had a negative direct effect on P_n_ (Figure 3b). The increase in tree size had a significant negative direct effect on G_s_ and SLA (Figure 3a). With the change in tree size, G_s_ had a direct effect on P_n_. ET and N not only have a direct effect on P_n_, but also have an indirect effect on P_n_ by directly affecting Gs (Figure 3a). The increase in tree size had a significant negative direct effect on G_s_, N and SLA (Figure 3b). SLA and G_s_ had a direct effect on P_n_, PT and N, not only has a direct effect on P_n_, but also has an indirect effect on P_n_ by directly affecting G_s_ (Figure 3b).

## 3. Discussion

### 3.1. Variations in Leaf Traits between Different Tree Size Groups or Shade-Tolerant Groups

Tree species had significant effects on all leaf traits (*p* < 0.05); species with strong shade tolerance tended to have lower P_n_, Gs and higher SLA (Figure S1, Table 2). The results showed that the photosynthetic capacity of different tree species was different, and photosynthetic capacity was related to shade tolerance. Similarly, according to the relationship between leaf traits of shade-tolerant species and shade-intolerant species in Table 1, as well as the relationship between leaf traits of different tree species in Figure S2, we can assume that the interspecific differences represented by different shade tolerance tree species in this study are similar to the interspecific variation based on genetic differences.

Consistent with our first hypothesis, there were significant differences in leaf traits among trees of different shade tolerances in this study (Table 1). Our study shows that shade tolerance is a good predictor of leaf anatomical traits. Most of the anatomical traits are significantly different between different shade-tolerant trees. Shade-intolerant species tend to have thicker PT and larger PT/LT (Table 1), which is in line with our expectations. Shade-intolerant trees live in a better light environment, and thicker PT can guide the leaves to better absorb light [46]. The PT/ST of shade-intolerant trees is higher, which is beneficial to better gas exchange in leaves [47], and can further enhance their photosynthetic ability. The shade-intolerant trees had lower ET than shade-tolerant trees. This represents a trade-off in the allocation of plant leaf resources. ET is often related to plant drought resistance [48,49]. Shade-intolerant trees allocate more resources to palisade tissue, which means that shade-intolerant species tend more towards obtaining resources rather than conservative growth. This growth strategy accordingly sacrifices part of the investment in the epidermis that helps to resist stress [9]. Therefore, when the water is sufficient, the shade-intolerant trees have higher carbon assimilation ability and can achieve faster growth, but when the drought is serious, the shade-tolerant trees have a stronger ability to resist water stress.

Similar to previous studies, the N and P of shade-intolerant trees were higher than those of shade-tolerant species (Table 1) in this study. This is because shade-intolerant trees are at the end of resource acquisition in LES. The higher N in the leaves, the greater the carbon gain without light restriction. Shade-tolerant trees are located at the conservative end of resource use. Compared with shade-intolerant trees, their maximum carbon yield is smaller, but they have better tolerance to light resource stress, so they have a larger niche [50]. The G_s_ of shade-intolerant trees is higher than that of shade-tolerant trees. There are three possible explanations: (1) The availability of N can affect the stomatal response. G_s_ in plant leaves usually increases with the increase in N content [51]; (2) G_s_ is anatomically regulated, and the PT/ST of leaves is inversely proportional to the number of intercellular spaces, so the higher the PT/ST of leaves, the more need for gas exchange, which will indirectly lead to the increase in G_s_ [47]; (3) The shade tolerance itself will affect the opening and closing of stomata. For example, trees with shade tolerance can open stomata in poor light conditions, and stomata can be opened faster under bright spots than shade-intolerant trees [52]. Therefore, we boldly predict that under the condition of light saturation, stomatal conductance decreases with the increase in shade tolerance, because shade-tolerant trees enhance their ability to absorb carbon dioxide in low light at the expense of maximum gas exchange capacity.

In contrast to LES theory, SLA in this study did not decrease with the increase in shade tolerance (Table 1). We think this may be because this study was conducted in broad-leaved deciduous species; in this case, lower SLA cannot increase the leaf life span of shade-tolerant trees, and the leaf-cost recovery time cannot be prolonged, so shade-tolerant species tend to choose lower quality leaf area in order to reduce the leaf construction cost [53].

The leaf N, P, PNUE and PPUE of trees of different sizes had significant differences (*p* < 0.05), and the four traits of small trees were significantly higher than those of large trees (Table 2). This result not only further verified the LES theory, but also showed that the photosynthetic capacity of trees at different growth stages not only depended on the changes of N and P content in leaves, but also on the utilization efficiency of nitrogen and phosphorus by the photosynthetic structure. Tree size had significant effects on ET, PT and ST (*p* < 0.05), but had no effect on PT/ST, PT/LT and ST/LT (Table 2), indicating that the anatomical trait of leaf thickness changed with the growth of trees, but its proportion in leaves was stable. In this study, the G_s_ of large trees was significantly lower than that of small trees (*p* < 0.05) (Table 2) Trees respond to changes in water availability by adjusting stomata [54]. According to the hydraulic limitation hypothesis, the hydraulic resistance increases with the increase in tree height, so large trees are more susceptible to drought than small ones [55,56]. To address this hydraulic limitation, large trees will reduce the G_s_ of leaves to form a compensation mechanism to prevent the plant from losing water too fast [36,37]. The compensation mechanism of large trees for water stress is also reflected in the morphological structure (SLA) (Table 1). To compensate for water stress, the leaves of large trees need to reduce the water potential to obtain enough water, so the leaves of tall trees may increase the investment of solutes such as starch and lipids, resulting in a decrease in SLA [57,58].

We found that the differences in the intraspecific and interspecific traits of leaves were driven by different strategies, and the differences of intraspecific traits among trees of different sizes were driven by SLA (Figure 1). Small trees showed a resource acquisition strategy with high SLA, while large trees tended to grow conservatively. The difference of interspecific traits among different shade-tolerant groups is driven by G_s_ (Figure 1). The shade-intolerant trees are in the stage of high carbon acquisition, while the shade-tolerant trees are at the carbon-conservative end. The coefficient of variation of each leaf trait also showed that, on the whole, the leaf traits of small trees and shade-intolerant species fluctuated greatly, while those of large trees and shade-tolerant species were more stable (Table S1).

### 3.2. Effects of Tree Size and Shade Tolerance on P_n_

Our study shows that the P_n_ of shade-tolerant species is lower than that of shade-intolerant species (Table 2). The photosynthetic capacity of plant leaves is mainly determined by two factors: (1) the biochemical carbon sequestration ability of leaves, and (2) the CO_2_ concentration in chloroplasts [12], in which the biochemical carbon sequestration capacity of leaves is mainly related to the maximum carboxylation rate, and the concentration of CO_2_ in chloroplasts is usually determined by the diffusion rate of CO_2_. In this study, the main leaf traits affecting the maximum carboxylation rate of leaves were PT, N and P, because these three traits could affect the content of chlorophyll and Rubisco in leaves, or directly participate in the process of photosynthesis [4,59]. The main leaf trait affecting CO_2_ diffusion rate was G_s_, because G_s_ is the main factor affecting gas exchange inside and outside leaves [24]. SLA affects these two factors at the same time; on the one hand, SLA is related to the percentage of cell wall in leaves, and leaves with low SLA usually have a large proportion of cell wall and stratum corneum, which increases the difficulty of carbon dioxide diffusion [5]. On the other hand, SLA reflects the trade-off between leaf mass and area, and higher SLA often indicates that leaves tend to use larger leaf area to obtain carbon resources [60]. For all types of trees, the contribution rate of G_s_ to P_n_ variation is the highest (Figure 2), indicating that the photosynthetic ability of plant leaves is mainly limited by the ability to obtain CO_2_. However, for different types of trees, the contribution of other leaf traits to the photosynthetic capacity of the tree is also different. The chemical traits of leaves make a greater contribution to the photosynthetic ability of small trees and shade-intolerant species, while the anatomical traits of leaves make a greater contribution to the photosynthetic capacity of large trees and shade-tolerant species (Figure 2). This is because small trees and shade-intolerant trees are at the end of resource acquisition of LES, and more nutrients are put into the leaves to promote the rapid growth of trees. Large trees and shade-tolerant trees are at the conservative end of resource acquisition and pay more attention to the investment in leaf toughness in order to obtain stronger resistance. The results of HP showed that the variation of P_n_ depended not only on the single leaf structure trait, but also was commonly limited by the morphological, chemical, anatomical and stomatal traits of leaves.

Shade tolerance of trees can directly affect leaf photosynthetic capacity, but also indirectly affect leaf photosynthetic capacity by affecting leaf structural traits (Figure 3b). Shade-intolerant trees can enhance G_s_, improve gas exchange capacity [61], increase the investment in leaf N, increase the maximum carboxylation rate of leaves [12], and further improve the efficiency of carbon sequestration. Shade-tolerant trees adopt a more conservative strategy of resource acquisition, devoting more resources to supporting organs such as roots and branches [62], in order to enhance the competitiveness of trees. The specific manifestation of this resource trade-off is that the N and PT of shade-tolerant trees are lower than that of shade-intolerant trees. Similarly, compared with small trees, large trees adopt a more conservative resource acquisition strategy, which reduces the area receiving light per unit mass (SLA) and carbon dioxide absorption capacity (G_s_) (Figure 3a), devotes more resources to non-photosynthetic tissues [31,32], reduces the growth rate of large trees, but improves the competitiveness of large trees.

Our study explains how shade tolerance and tree size affect tree photosynthesis indirectly by affecting leaf structure traits on a local scale, which makes up for the data gaps in related fields and provides new evidence for the relationship between tree traits. However, it is undeniable that all our experiments were conducted on a limited number of tree species in broad-leaved Korean pine forests in northern China, which means limitations, because leaf traits of trees usually vary with different plant functional groups or environmental conditions [19,45]. In future research, we hope to make up for the deficiency of the current research through more experiments.

## 4. Materials and Methods

### 4.1. Research Site

All research was performed in the Liangshui National Nature Reserve (47°6′~47°16′N, 128°47′~128°57′E) in Heilongjiang Province, Northeast China. The site is a hilly region with a temperate continental monsoon climate. The mean annual temperature is −3 °C, and the mean annual precipitation and evaporation are 676 mm and 805 mm, respectively. The zonal soil of the region is dark brown forest soil, and the zonal vegetation of the region is mixed broad-leaved Korean pine (*Pinus koraiensis*) forest, and the dominant tree species are *Pinus koraiensis*, *Abies nephrolepis*, *Acer pictum* subsp. *mono*, *Betula platyphylla* and *Fraxinus mandschurica* [63,64].

### 4.2. Sampling

Six major broadleaf species were selected at the sampling site, including *Acer pictum* subsp. *mono*, *Acer tegmentosum*, *Betula platyphylla*, *Fraxinus mandschurica*, *Juglans mandshurica* and *Ulmus laciniata*. Six tree species were divided into two types (shade-tolerant trees and shade-intolerant trees) based on their relative shade tolerance, among which shade-tolerant trees included *Acer pictum* subsp. *mono*, *Acer tegmentosum*, *Ulmus laciniata*, and shade-intolerant trees included *Betula platyphylla*, *Fraxinus mandschurica*, *Juglans mandshurica* [65,66]. Each tree species was divided into two sizes based on tree height (these trees are of different ages, so they are of different sizes). For each species and each size, ten individuals were randomly sampled in August 2021, including 7 small individuals from *Juglans mandshurica*, for a total of 117 sample trees (the basic traits of the trees are shown in Table 3). The sampling lasted for a total of 12 days and was only taken on sunny days from 8 to 11 o **‘**clock. All trees were sampled in the upper canopy on the sunny side. The mature leaves were collected from the sunny side of each sample tree for the determination of photosynthetic rate. The light intensity of the Li-6400 (LI-COR, Lincoln, USA) photosynthesis system was set to 1500 (μmol·m**^−^**^2^·s**^−^**^1^), and the concentration of CO_2_ was set to 400 (μmol mol**^−^**^1^). According to past experience, under these conditions, the leaf can reach the maximum net photosynthetic rate [6]. Three leaves of each tree were selected to measure the area-based maximum net photosynthetic rate (P_n-area_, μmol m**^−^**^2^ s**^−^**^1^) and stomatal conductance (G_s-area_, μmol m**^−^**^2^ s**^−^**^1^). Photosynthetic traits were measured in the field. The leaves with measured photosynthetic traits were kept fresh and sent to the laboratory as soon as possible to measure their morphological traits. Ten leaves were selected from each individual and preserved in formalin-acetic acid-alcohol (FAA) solution for the analyses of anatomical traits [67]. The rest of the leaves were dried to constant weight in a baking oven at 65 ℃ and were used to measure chemical properties.

### 4.3. Leaf Trait Measures

#### 4.3.1. Leaf Morphological Traits

The leaves were scanned into pictures by a Canon LiDE 400 scanner (Canon Inc., Tokyo, Japan), and the leaf area calculation program was used to calculate the leaf area (LA, cm^2^) through the pixels of the picture. Then the leaf samples were dried in a baking oven at 65 ℃ to constant weight, and the leaf dry weight (DW, g) was measured with an electronic balance (accuracy 0.0001 g). The formula for calculating LMA (g m**^−^**^2^) and SLA (m^2^ g^-1^) are as follows:

LMA = 10,000 × DM/LA(1)

SLA = 1/LMA(2)

The formula for converting leaf P_n-area_ and G_s-area_ into mass-based maximum net photosynthetic rate (P_n_, μmol g**^−^**^1^ s**^−^**^1^) and stomatal conductance (Gs, μmol g**^−^**^1^ s**^−^**^1^) is as follows:

P_n_ = P_n-area_/LMA (3)

G_s_ = G_s-area_/LMA (4)

#### 4.3.2. Leaf Chemical Traits

Leaf samples used for measuring chemical traits were oven-dried to constant weight at 65 °C for grinding. The total nitrogen content (N, mg g**^−^**^1^) and total phosphorus content (P, mg g**^−^**^1^) of leaf samples were measured by CleverChem380 (DeChem-Tech. GmbH, Hamburg, Germany) automatic discontinuous chemical analyzer after H_2_SO_4_-H_2_O_2_ digestion. The total carbon contents (C, mg g**^−^**^1^) of leaf samples were measured by multiN/C3000 (Analytik Jena AG, Jena, Germany) carbon and nitrogen element analyzer.

The formulas for calculating the utilization efficiency of photosynthetic nitrogen (PNUE) and phosphorus (PPUE) are as follows:

PNUE = P_n_/N (5)

PPUE = P_n_/P (6)

#### 4.3.3. Leaf Anatomical Traits

Three intact leaves per individual were selected from the FAA solution, and the leaf sections with a thickness of 6 μm were obtained by the paraffin section technique and stained [9]. The leaf sections were observed by a light microscope (Olympus Electronics, Inc., Tsukuba, Japan), and photographed by cellSens Standard 1.11 software (Olympus Electronics Inc., Tsukuba, Japan) and measured by ImageJ 1.53a software (National Institutes of Health, Bethesda, USA). Three photographs were selected per leaf section for measuring the adaxial epidermis thickness (AD, μm), abaxial epidermis thickness (AB, μm), PT (μm) and ST (μm). The summed value of AT and AB was taken as epidermis thickness (ET, μm).

### 4.4. Data Analysis

An independent samples t-test in SPSS 21.0 was applied to examine the differences in leaf traits between shade-tolerant trees and shade-intolerant trees. The influence of tree species and tree sizes on leaf traits were tested by two-factor analysis of variance, and the influence of tree species on leaf traits was tested after the fact. The following data analyses were carried out in R 4.2.1. Principal component analysis was used to analyze the relationship among leaf traits. The ‘hier.part’ package was used to perform hierarchical partitioning (HP) analysis of leaf traits and quantify the explanation rate of different traits to P_n_ [68]. The effect of shade tolerances and tree sizes and leaf traits on P_n_ were studied by structural equation modeling (SEM) constructed by the **‘**lavaan**’** package [69]. All traits were log-transformed before calculation.

## 5. Conclusions

With the increase in shade tolerance of tree species or tree size, the net photosynthetic rate of tree leaves decreased. This variation is not determined by single leaf traits, but caused by the differences of many leaf traits. Generally speaking, the N, P (or their use efficiency) and G_s_ in the leaves of shade-intolerant species or small trees are higher, which means that shade-intolerant species or small trees have more nutrients to provide for photosynthesis, and at the same time, the resistance of CO_2_ diffusion in leaves is lower, which is helpful to improve the photosynthetic capacity of leaves. At the same time, the leaves of shade-tolerant trees usually have higher epidermal tissue thickness, although the photosynthetic ability of plants is further reduced, but the greater leaf toughness makes their leaves better resistant to external water stress and physical damage, and enhance their competitiveness. Therefore, the shade tolerance of tree species and tree sizes plays a key role in the variation of leaf photosynthetic capacity, and should be considered in the study of leaf photosynthetic capacity.

## Figures and Tables

**Table 1:** T-test results of leaf traits between shade-intolerant trees and shade-tolerant tree groups.

Leaf Traits	Shade-Tolerant Groups		Leaf Traits	Shade-Tolerant Groups	
	T	*p*-Value		T	*p*-Value
P_n_	7.387	**<0.001**	PT/LT	13.756	**<0.001**
SLA	−3.901	**<0.001**	ST/LT	−5.598	**<0.001**
Gs	5.331	**<0.001**	C	9.410	**<0.001**
ET	−4.379	**<0.001**	N	3.504	**0.001**
PT	11.435	**<0.001**	P	8.375	**<0.001**
ST	−0.036	0.971	PNUE	5.687	**<0.001**
PT/ST	9.572	**<0.001**	PPUE	3.620	**<0.001**

Bold indicates a significant difference (*p* < 0.05). P_n_, maximum net photosynthetic rate based on mass; ET, epidermis thickness; PT, palisade tissue thickness; ST, spongy tissue thickness; PT/ST, palisade–spongy tissue ratio; PT/LT, palisade tissue–leaf thickness ratio; ST/LT, spongy tissue–leaf thickness ratio; C, carbon content; N, nitrogen content; P, phosphorus content; PNUE, utilization efficiency of photosynthetic nitrogen; PPUE, utilization efficiency of photosynthetic phosphorus; (PPUE) SLA, specific leaf area; G_s_, stomatal conductance based on mass.

**Table 2:** Effect of species and tree size on leaf traits.

Source	P_n_		SLA		G_s_	
	F	P	F	P	F	P
Species	34.664	**<0.001**	20.054	**<0.001**	15.391	**<0.001**
Tree size	87.059	**<0.001**	48.959	**<0.001**	49.800	**<0.001**
Species × Tree size	6.868	**<0.001**	7.844	**<0.001**	7.454	**<0.001**
	**ET**		**PT**		**ST**	
	**F**	**P**	**F**	**P**	**F**	**P**
Species	29.440	**<0.001**	79.129	**<0.001**	70.469	**<0.001**
Tree size	9.296	**0.003**	14.469	**<0.001**	17.348	**<0.001**
Species × Tree size	2.043	0.079	3.077	**0.012**	2.704	**0.024**
	**PT/ST**		**PT/LT**		**ST/LT**	
	**F**	**P**	**F**	**P**	**F**	**P**
Species	137.328	**<0.001**	126.407	**<0.001**	108.730	**<0.001**
Tree size	0.133	0.716	0.270	0.604	1.476	0.227
Species × Tree size	0.471	0.797	0.426	0.830	1.043	0.396
	**C**		**N**		**P**	
	**F**	**P**	**F**	**P**	**F**	**P**
Species	155.950	**<0.001**	4.792	**0.001**	26.268	**<0.001**
Tree size	18.293	**<0.001**	4.074	**0.046**	8.894	**0.004**
Species × Tree size	1.058	0.388	3.761	**0.004**	4.505	**0.001**
	**PNUE**		**PPUE**			
	**F**	**P**	**F**	**P**		
Species	16.893	**<0.001**	8.050	**<0.001**		
Tree size	46.159	**<0.001**	40.123	**<0.001**		
Species × Tree size	3.647	**0.004**	1.807	0.118		

Bold indicates a significant difference (*p* < 0.05). P_n_, maximum net photosynthetic rate based on mass; ET, epidermis thickness; PT, palisade tissue thickness; ST, spongy tissue thickness; PT/ST, palisade–spongy tissue ratio; PT/LT, palisade tissue–leaf thickness ratio; ST/LT, spongy tissue–leaf thickness ratio; C, carbon content; N, nitrogen content; P, phosphorus content; PNUE, utilization efficiency of photosynthetic nitrogen; PPUE, utilization efficiency of photosynthetic phosphorus; (PPUE) SLA, specific leaf area; G_s_, stomatal conductance based on mass.

**Table 3:** Means and standard errors (SE) of basic traits of trees in mixed broad-leaved Korean pine forests.

Species	Small Tree		Large Tree	
	DBH (cm)	H (m)	DBH (cm)	H (m)
All species	3.16 ± 0.12	4.11 ± 0.16	26.15 ± 1.19	19.18 ± 0.63
*Acer pictum* subsp. *mono*	2.74 ± 0.27	4.04 ± 0.32	24.22 ± 1.98	16.76 ± 1.16
*Acer tegmentosum*	3.1 ± 0.35	3.92 ± 0.15	14.20 ± 1.25	13.13 ± 1.01
*Ulmus laciniata*	2.58 ± 0.12	3.38 ± 0.21	26.18 ± 3.05	16.75 ± 0.61
*Betula platyphylla*	4.05 ± 0.16	5.40 ± 0.43	28.47 ± 2.25	21.00 ± 0.95
*Fraxinus mandschurica*	2.75 ± 0.27	3.77 ± 0.44	33.14 ± 2.60	24.19 ± 1.22
*Juglans mandshurica*	3.89 ± 0.37	4.36 ± 0.52	31.22 ± 2.20	23.62 ± 0.88

DBH: diameter at breast height of tree; H: height of tree.

## Data Availability

The data presented in this study are available upon request from the corresponding author. The data are not publicly available due to privacy reasons.

## Supplementary Materials

The following supporting information can be downloaded at: https://www.mdpi.com/article/10.3390/plants12030523/s1, Table S1: Basic information table for leaf traits; Figure S1: Variation in leaf traits between different tree species.
