# Supplementary material for: Do Tree Size and Tree Shade Tolerance Affect the Photosynthetic Capacity of Broad-Leaved Tree Species?
Source: Plants (Basel). 2023 Jan 23;12(3):523. doi: 10.3390/plants12030523 (PMC9921863; doi:10.3390/plants12030523)
Supplement: Supplementary file 1 [file plants-12-00523-s001.zip › plants-2161035-supplementary.pdf]

Table S1 Basic information table for leaf traits

Leaf traits	Mean value	Minimum value	Maximum value
P_n	0.19	0.07	0.49
ET	26.93	16.81	40.62
PT	49.44	25.58	103.93
ST	50.09	18.94	84.16
PT/ST	1.04	0.49	2.40
PT/LT	0.39	0.24	0.56
ST/LT	0.39	0.22	0.53
C	429.57	352.10	493.20
N	19.96	13.25	34.65
PNUE	0.0095	0.0032	0.0227
P	2.04	1.28	4.01
PPUE	0.0921	0.0403	0.2225
SLA	0.0250	0.0120	0.0471
G_s	0.0043	**0.0008**	0.0261

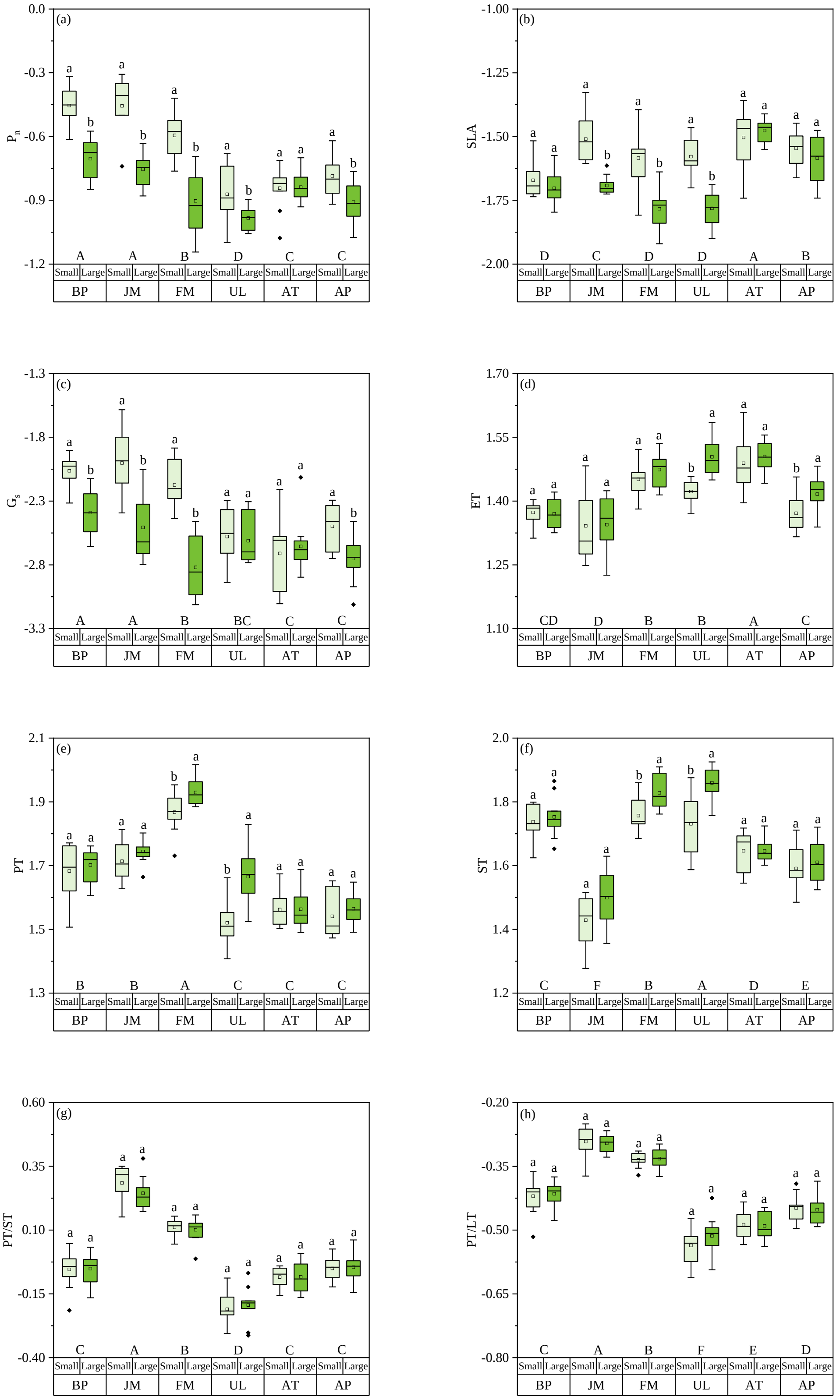
(a)
P_n
0.0
-0.3
-0.6
-0.9
-1.2
(b)
SLA
-1.00
-1.25
-1.50
-1.75
-2.00
(c)
G_s
-1.3
-1.8
-2.3
-2.8
-3.3
(d)
ET
1.70
1.55
1.40
1.25
1.10
(e)
PT
2.1
1.9
1.7
1.5
1.3
(f)
ST
2.0
1.8
1.6
1.4
1.2
(g)
PT/ST
0.60
0.35
0.10
-0.15
-0.40
(h)
PT/LT
-0.20
-0.35
-0.50
-0.65
-0.80
Small
Large
BP
JM
FM
UL
AT
AP

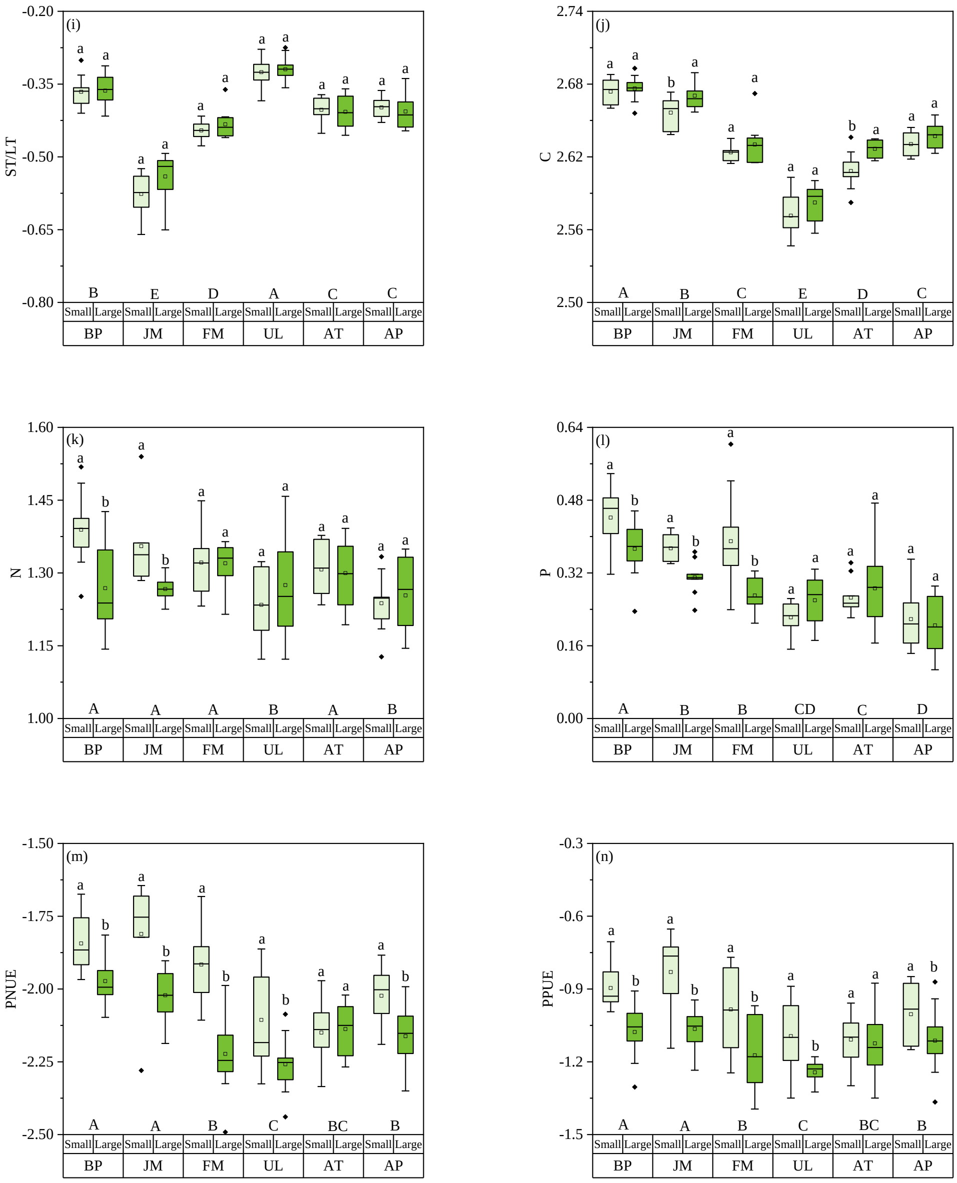

Figure S1 Variation in leaf traits between different tree species. Different capital letters indicated significant differences in leaf traits among different tree species (p < 0.05). Different lowercase letters indicate significant differences in leaf traits between different sizes of the same tree species (p < 0.05). The shade tolerance of tree species increased from left to right. BP: *Betula platyphylla*; JM: *Juglans mandshurica*; FM: *Fraxinus mandschurica*; UL: *Ulmus laciniata*; AT: *Acer tegmentosum*; AP: *Acer pictum* subsp. *mono*.
